# Role of Oxidants in Interstitial Lung Diseases: Pneumoconioses, Constrictive Bronchiolitis, and Chronic Tropical Pulmonary Eosinophilia

**DOI:** 10.1155/2011/407657

**Published:** 2011-10-30

**Authors:** William N. Rom

**Affiliations:** Division of Pulmonary, Critical Care, and Sleep Medicine, Departments of Medicine and Environmental Medicine, New York University School of Medicine, New York, NY 10016, USA

## Abstract

Oxidants such as superoxide anion, hydrogen peroxide, and myeloperoxidase from activated inflammatory cells in the lower respiratory tract contribute to inflammation and injury. Etiologic agents include inorganic particulates such as asbestos, silica, or coal mine dust or mixtures of inorganic dust and combustion materials found in World Trade Center dust and smoke. These etiologic agents are phagocytosed by alveolar macrophages or bronchial epithelial cells and release chemotactic factors that recruit inflammatory cells to the lung. Chemotactic factors attract and activate neutrophils, eosinophils, mast cells, and lymphocytes and further activate macrophages to release more oxidants. Inorganic dusts target alveolar macrophages, World Trade Center dust targets bronchial epithelial cells, and eosinophils characterize tropical pulmonary eosinophilia (TPE) caused by filarial organisms. The technique of bronchoalveolar lavage in humans has recovered alveolar macrophages (AMs) in dust diseases and eosinophils in TPE that release increased amounts of oxidants in vitro. Interestingly, TPE has massively increased eosinophils in the acute form and after treatment can still have ongoing eosinophilic inflammation. A course of prednisone for one week can reduce the oxidant burden and attendant inflammation and may be a strategy to prevent chronic TPE and interstitial lung disease.

## 1. Introduction

Gaseous and particulate air pollutants activate inflammatory cells to release oxidants such as superoxide anion, hydrogen peroxide, and hydroxyl radicals [1]. Oxidants damage proteins, lipids, DNA forming nitrotyrosine, 4-OH-2-nonenal, and 8-OHdeoxyguanosine, and single strand breaks [2]. Oxidants from macrophages and bronchial epithelial cells cause inflammation leading to injury and fibrosis [3]. These events are particularly important to the upper airways leading to asthma and constrictive bronchiolitis and to the lower respiratory tract where exogenous oxidants can activate macrophages to release endogenous oxidants, attract other inflammatory cells, and injure the delicate walls of the peripheral respiratory bronchioles and alveolar sacs [4]. The respiratory tract is a target organ for cigarette smoke, inorganic dusts (asbestos, silica, and coal), PM_2.5_, and World Trade Center dust contributing to asthma, COPD, fibrosis, and constrictive bronchiolitis [5]. Oxidants in the lung can be from endogenous sources since air pollutants activate neutrophils, alveolar macrophages, eosinophils, and epithelial cells. Antioxidants include superoxide dismutase and glutathione peroxidase.

Oxidants generate airway inflammation by activating NF-*κ*B and derepressing C/EBP*β* in macrophages leading to the production of proinflammatory cytokines IL-1*β*, TNF-*α*, IL-8, and IL-6 [6]. Oxidants are a defense mechanism against pathogens which adds to the amount of oxidative stress. Cigarette smoking contributes to oxidative stress directly and stimulates the release of chemokines to attract inflammatory cells [7].

## 2. Inorganic Dusts

Occupational exposures to coal, silica, or asbestos are the major causes of the pneumoconiosis or dust-induced lung diseases [8]. Exposure duration is important with most of these disorders requiring >10 years and usually >20 years before these diseases manifest. They continue to progress despite removal from exposure. Chest radiographs show small irregular or rounded opacities in increasing profusion, and profusion correlates with length of exposure. These opacities may become congruent increasing to 1-2 centimeters or more leading to complicated pneumoconiosis. Complicated pneumoconiosis or progressive massive fibrosis frequently leads to respiratory impairment. Symptoms are mostly shortness of breath with exertion or climbing stairs although cough, sputum production, and chest pain may also occur. Intensity of exposure is also important since workers directly exposed are at greatest risk. These include coal miners at the face, silica-exposed workers grinding sand-covered metal or polishing stone, and asbestos workers applying and cutting insulation or asbestos-formed products [9]. Pulmonary function tests may show a reduction of lung volumes consistent with pulmonary fibrosis and/or airways obstruction illustrating dust-related airway irritation and fibrosis. Smoking is common among this blue-collar workforce which interacts with dust exposure in causing respiratory symptoms, changes on the chest X-ray, and, especially, changes in pulmonary function tests. Phagocytosis of particles by alveolar macrophages (AMs) deposited in the respiratory bronchioles and alveolar ducts and sacs can activate AM and stimulate the release of oxidants including superoxide anion and hydrogen peroxide [10, 11]. Measurements of these two in short-term assays of freshly recovered bronchoalveolar lavage cells have noted increased levels in patients with coal worker's pneumoconiosis, asbestosis, and silicosis [10, 12]. Levels are increased in nonsmokers and those who have quit smoking for >5 years, and higher levels are noted in those with respiratory impairment compared to normal nonsmoking volunteers [13]. Age does not appear to be a confounder in these assays. Also, the predominant cell recovered is the AM although there are significant increases in neutrophils and lymphocytes consistent with a macrophage alveolitis. Measurements in 24-hour supernatants from bronchoalveolar lavage cells using immunoassays show increases in fibronectin compared to nonsmoking controls and increases in peptide growth factors including platelet-derived growth factor (PDGF), insulin-like growth factor (IGF-I), and transforming growth factor-beta (TGF-*β*) [14, 15]. TGF-*β* has been shown to be increased using immunohistochemistry of lung biopsies from workers with chronic or acute silicosis [16]. Cytokines are also increased in BAL supernatants including IL-6, IL-8, IL-1*β*, and TNF-*α*, and Northern analysis has shown the mechanism to be upregulation of their respective mRNAs [17, 18]. AMs have a unique transcriptional regulatory machinery since transcription factors that regulate inflammatory cytokines must keep the lower respiratory track in a state of downregulation in normal homeostasis. During the course of inflammation, these transcription factors need to be regulated to the “on” position for inflammatory cytokines to be produced and released. Interestingly, the four major inflammatory cytokines IL-1*β*, IL-6, IL-8, and TNF-*α* all have NF-*κ*B and C/EBP*β* sites in their promoters [19]. During normal homeostasis, NF-*κ*B is bound to I*κ*B and not activated in the nucleus. C/EBP*β* is expressed primarily in the short 16 kDa form which is inhibitory to transcription at low levels of protein even at ratios of 1 : 5 with the 36 kDa activating form [20]. During AM activation, the inhibitory form is de-repressed and the activating form predominates. NF-*κ*B is released from I*κ*B and binds to its cognate DNA binding site [21]. The combination of derepression and activation provides a powerful signal for AM activation [22]. Oxidants are triggers for this process upon phagocytosis of inorganic dust particles. 

## 3. World Trade Center Dust

Nuisance dusts have not been regulated since they do not cause a pneumoconiosis. The collapse of the World Trade Center produced a huge cloud of nuisance dust containing gypsum from wallboard, plastics, cement, fibrous glass, asbestos insulation, metals, and so forth. This acute, intense dust exposure has resulted in a remarkable increase in symptoms characterized by cough, wheeze, chest tightness, gastroesophageal reflux, and so forth in firefighters, rescue workers, and clean-up workers. Residents who returned to their apartments and cleaned up the dust have also been affected [23]. Asthma has been found to have been worsened, and there is an increase in newly diagnosed asthma. Pulmonary function tests have found simultaneous reductions in FVC and FEV_1_ that have been several-fold greater than expected. Significant declines in FEV_1_ were noted shortly after the collapse. Pulmonary function tests of peripheral airways function have been abnormal despite normal spirometry [24]. Smoke from persistent fires containing polycyclic aromatic hydrocarbons, metals, and many other chemicals exposed those involved in rescue and clean-up operations.

Two weeks after the collapse we admitted a severely dyspneic firefighter to Bellevue Hospital who was in acute respiratory failure [25, 26]. His chest X-ray showed bilateral pulmonary infiltrates and pleural effusions. Following treatment with steroids and antibiotics he improved. A bronchoalveolar lavage found 70% eosinophils in the differential, and a diagnosis of acute eosinophilic pneumonia was made. He had increased IL-5, an eosinophil chemoattractant, in the BAL supernatants. Interestingly, his BAL eosinophils showed intact granules containing major basic protein on transmission electron microscopy. This suggested that the eosinophils although responding to a strong chemoattractant signal had not become activated to release their granular contents. This was consistent with the patient's benign course and complete recovery from the pneumonia. However, the firefighter continued to complain of World Trade Center cough for several more months [27, 28]. 

## 4. Tropical Pulmonary Eosinophilia (TPE)

Oxidants play a key role in tropical pulmonary eosinophilia in causing both an acute disease and chronic interstitial pulmonary fibrosis. The eosinophil is central to this process, and, in contrast to acute eosinophilic pneumonia, the granules are empty and major basic protein has been extruded [29]. This contributes to high oxidant stress in the terminal respiratory bronchioles and alveoli. TPE is an interstitial lung disease that results from a heightened immunologic response to the human filarial parasites, *Wuchereria bancrofti* and *Brugia malayi. * Individuals with acute TPE characteristically present with cough, dyspnea, nocturnal wheezing, and, occasionally, fever, anorexia, and weight loss. It occurs in both males and females in the younger age groups in India, Southeast Asia, and other tropical regions. Filariasis is spread by mosquitoes, but only a small portion of the population responds with the TPE syndrome. There are a marked peripheral blood eosinophilia (in contrast to acute eosinophilic pneumonia where our firefighter had a normal blood eosinophil count) and high serum concentrations of IgE and filarial specific antibodies. Although most individuals with acute TPE have a rapid clinical response to diethylcarbamazine or ivermectin, with reduced cough and dyspnea, some individuals progress to a chronic pulmonary fibrosis. It is thought that the pulmonary form of TPE results from degenerating microfilariae in the pulmonary structures, but few have been found in lung specimens, suggesting that most of the pathology seen and symptoms arise from the lung inflammation and oxidants. 

In a clinical study of TPE in India conducted by a joint Indian Council of Medical Research and a NIH research team approved by Human Subjects Review utilizing bronchoalveolar lavage, eight individuals were bronchoscoped and a mean of 54% eosinophils recovered in BAL [29]. Transmission EM showed marked loss of granule content and disappearance of dense central cores. Two of three individuals bronchoscoped one year later still had marked BAL eosinophilia, although reduced [29]. 

**Figure 1:**
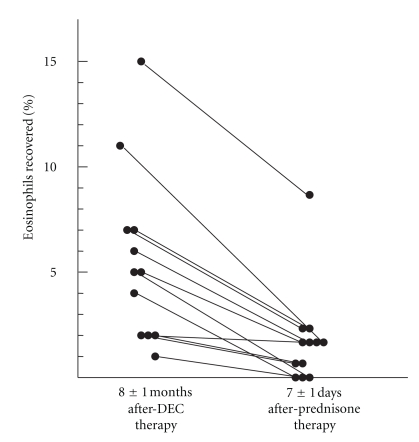
Suppression of lung eosinophils with prednisone in TPE patients following after diethylcarbamazine therapy. 7±1 days after prednisone therapy there was a reduction in the percent of eosinophils observed in 12 subjects.

**Figure 2:**
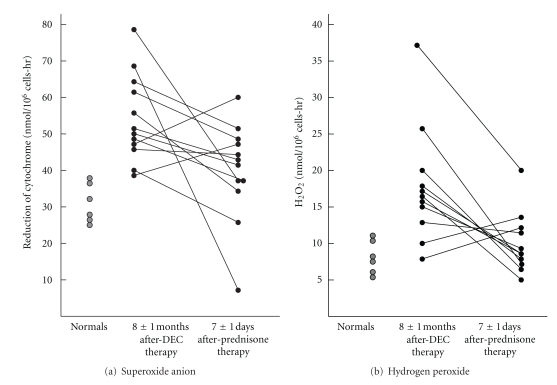
Reduction in the spontaneous release of oxidants by lavage cells after prednisone therapy in TPE patients following diethylcarbamazine therapy. (a) Superoxide anion. (b) Hydrogen peroxide.

One year later, 23 subjects who had been treated were reevaluated [30]. They had a mean age of 26 years, and 15 still complained of cough and nocturnal wheezing. Only 3/20 had dyspnea, chest pain, or rales. They still had tenfold increased IgE and IgG antifilarial antibodies, but much less than when first studied [30]. Their BAL had a mean of 6% eosinophils. There was a significant spontaneous increase in superoxide anion and hydrogen peroxide release by the BAL cells over 30 minutes following recovery in twenty chronic TPE subjects (6 smokers, 14 nonsmokers) compared to six normal nonsmokers a mean of 8 months following diethylcarbamazine therapy [30]. In order to reduce the inflammation in the lower respiratory track, a short course of oral prednisone over one week was attempted. The dose was 50 mg followed by a daily 10 mg decline until they were off medication. Bronchoalveolar lavage was performed prior to the beginning of the trial and immediately thereafter. Twelve individuals completed the trial, and the BAL percent eosinophils declined or remained the same in all study subjects after a mean of seven days of oral prednisone (Figure 1). Measurements of superoxide anion and hydrogen peroxide were done on fresh BAL cells recovered by bronchoscopy and superoxide anion declined in 10/12 study subjects (Figure 2). In parallel, hydrogen peroxide declined in 9/11 study subjects. Several clinical series from India, Southeast Asia, Africa, the West Indies, and South America have described untreated TPE as a cause of chronic interstitial lung disease [31]. In this regard, it is recognized that if acute TPE is allowed to persist for more than 6 months without treatment, restrictive lung impairment is common. Open lung biopsies of individuals with TPE untreated for >6 months reveal infiltration of the alveolar structures and bronchiolar walls, with macrophages and eosinophils together with granulomata laden with eosinophils and multinucleated giant cells and interstitial fibrosis. The role of oxidants in causing interstitial lung disease in chronic TPE is significant, and this inflammatory milieu can be modulated by anti-inflammatory treatment with prednisone or ivermectin [32].

## 5. Summary

Air pollutants and oxidants can attract and activate inflammatory cells and signaling pathways which promote inflammation. Inhibition of inflammatory pathways with corticosteroids such as c-Jun terminal kinase pathway in mice attenuated O_3_-induced inflammation and hyperresponsiveness [33]. Oxidants increase NF-*κ*B DNA binding along with the release of IL-8 in lung epithelial cells, and this effect can be abrogated by antioxidant pretreatment [34]. Particulate-matter- (PM-) induced activation of NF-*κ*B has been shown to be dependent on the mitogen-activated protein kinase signaling pathway, which is involved in the stress response [35]. Nrf2 is another important transcription factor that binds to antioxidant response elements leading to the induction of various genes involved in mitigating oxidant damage [36]. Particulate matter less than 2.5 microns in air pollution and inorganic particles activate AM to release superoxide anion and hydrogen peroxide and cause DNA damage. Particles that cause DNA damage may also be involved in lung carcinogenesis [37].
